# Time-dependent variation of pathways and networks in a 24-hour window after cerebral ischemia-reperfusion injury

**DOI:** 10.1186/s12918-015-0152-4

**Published:** 2015-02-27

**Authors:** Li-Ying Wang, Jun Liu, Yuan Li, Bing Li, Ying-Ying Zhang, Zhi-Wei Jing, Ya-Nan Yu, Hai-Xia Li, Shan-Shan Guo, Yi-Jun Zhao, Zhong Wang, Yong-Yan Wang

**Affiliations:** Institute of Basic Research in Clinical Medicine, China Academy of Chinese Medical Sciences, Dongzhimennei Nanxiaojie 16#, Beijing, 100700 China; Beijing University of Chinese Medicine, No. 11 East Road, North of 3rd Ring Road, Beijing, 100029 China; Institute of Information on Traditional Chinese Medicine, China Academy of Chinese Medical Sciences, Dongzhimennei Nanxiaojie 16#, Beijing, 100700 China; Guang’anmen Hospital, China Academy of China Medical Sciences, No.5 Beixiange, Beijing, 100053 China; China Academy of Chinese Medical Sciences, Dongzhimennei Nanxiaojie 16#, Beijing, 100700 China; Institute of Chinese Materia Medica, China Academy of Chinese Medical Sciences, Dongzhimennei Nanxiaojie 16#, Beijing, 100700 China

**Keywords:** Ischemia-reperfusion injury, Dynamic variation, Pathway, Process network

## Abstract

**Background:**

Cerebral ischemia-reperfusion injury may simultaneously result in functional variation of multiple genes/pathways. However, most prior time-sequence studies on its pathomechanism only focused on a single gene or pathway. Our study aimed to systematically analyze the time-dependent variation in the expression of multiple pathways and networks within 24 h after cerebral ischemia-reperfusion injury.

**Results:**

By uploading 374 ischemia-related genes into the MetaCore software, the variation in the expression of multiple pathways and networks in 3 h, 12 h, and 24 h after cerebral ischemia-reperfusion injury had been analyzed. The conserved TNFR1-signaling pathway, among the top 10 pathways, was consistently enriched in 3 h, 12 h, and 24 h groups. Three overlapping pathways were found between 3 h and 12 h groups; 2 between 12 h and 24 h groups; and 1 between 3 h and 24 h groups. Five, 4, and 6 non-overlapping pathways were observed in 3 h, 12 h, and 24 h groups, respectively. Apart from pathways reported by earlier studies, we identified a novel pathway related to the time-dependent development of cerebral ischemia pathogenesis. The process of apoptosis stimulation by external signals, among the top 10 processes, was consistently enriched in 3 h, 12 h, and 24 h groups; 2, 1, and 2 processes overlapped between 3 h and 12 h groups, 12 h and 24 h groups, and 3 h and 24 h groups, respectively. Four, 5, and 5 non-overlapping processes were found in 3 h, 12 h and 24 h groups, respectively. The presence of apoptotic processes was observed in all the 3 groups; while anti-apoptotic processes only existed in 3 h and 12 h groups. Additionally, according to node degree, network comparison identified 1, 8,and 5 important genes or proteins (e.g. Pyk2, PKC, E2F1, and VEGF-A) in 3 h, 12 h, and 24 h groups, respectively. The Jaccard similarity index revealed a higher level of similarity between 12 h and 24 h groups than that between 3 h and 12 h groups.

**Conclusion:**

Time-dependent treatment can be utilized to reduce apoptosis, which may activate anti-apoptotic pathways within 12 h after cerebral ischemia-reperfusion injury. Pathway and network analyses may help identify novel pathways and genes implicated in disease pathogenesis.

**Electronic supplementary material:**

The online version of this article (doi:10.1186/s12918-015-0152-4) contains supplementary material, which is available to authorized users.

## Background

A series of pathological changes appear successively within 24 h after cerebral ischemia-reperfusion injury. These include pyknosis, degeneration, and edema of neurons and glial cells within 3 h, with cell death and edema peaking at 24 h [1]. These changes were significantly correlated with factors like excessive free radical formation [2], blood–brain barrier (BBB) damage [3,4], amino acid excitotoxicity [5], inflammatory response [6], and intracellular calcium overload [7,8].

In recent years, several reports have examined the time-course of the development of cerebral ischemia, so as to identify the underlying mechanisms. For example, ubiquitin + 1 immunoreactivity markedly increased 2 days after ischemia-reperfusion (I-R), but became very weak 5 days after I-R [9]. The expression level of AQP-4 decreased quickly after focal cerebral I-R injury, reaching its lowest level 12–24 h after reperfusion; then it increased slowly, returning to its normal level 7 days after reperfusion [10]. MMP-9 and gp91phox expression levels were up-regulated in the ischemic hemisphere of the brain tissue after 90 min of MCAO with 22.5 h of reperfusion [11]. Glucokinase (GK) and glucokinase regulatory protein (GKRP) immunoreactivities in the pyramidal neurons were distinctively decreased in the hippocampal CA1 region, but not in CA2/3, 3 days after I-R. Five days after I-R, GK and GKRP immunoreactivities were hardly detectable in the CA1, but not in CA2/3, pyramidal neurons; however, at this point in time, GK and GKRP immunoreactivities were detected in astrocytes, not microglia, in the ischemic CA1 [12]. The level of Vegfa mRNA increased in the rat brain, 4 h after occlusion in the cerebellum, cerebral cortex, and hippocampus, after 8 h in the cortex and hippocampus, and after 24 h in the cortex [13].

Previous studies mostly focused on the role of a single molecule or protein. Due to the multifaceted pathomechanism of cerebral ischemia, it is unlikely that a single indicator is sufficient to influence the outcomes. Thus, a study on the time-dependent pathological mechanism evaluating the role of multiple molecules/proteins in cerebral ischemia is required. Our previous study probed the dynamic pathological mechanism and found several important genes and pathways within 24 h after cerebral I-R by analyzing the gene expression profiles of ischemia-reperfusion and pathways from the KEGG database at 3 h, 12 h, and 24 h in the hippocampus of ischemic mice [14]. However, that study paid more attention to individual genes and less attention to the pathways and networks where these genes were not involved. The current study analyzes the mechanism of the pathological changes within 24 h after cerebral ischemia-reperfusion injury in the context of pathways and networks of protein molecules. The GeneGo MetaCore^TM^ software, with its high-quality and manually-curated databases, was used to integrate and visualize the data to glean information about signaling and metabolic pathways and the effects of bioactive molecules [15-17].

## Methods

### Animal model

Animal experiments were carried out in accordance with the Prevention of Cruelty to Animals Act (1986) and the National Institute of Health Guideline on the Care and Use of Laboratory Animals for experimental procedures. The experimental protocol was approved by the Ethics Review Committee for Animal Experimentation, China Academy of Chinese Medical Sciences. One hundred and eighty healthy adult male Kunming mice, at 12 weeks of age (weight 38–48 g), were obtained from the Experimental Animal Center in Health Science Center of Peking University. They were randomly assigned into 4 groups: sham, ischemia-reperfused 3 h, ischemia-reperfused 12 h, and ischemia-reperfused 24 h. In the ischemia-reperfused 3 h, 12 h and 24 h groups, according to Hara’s method [18], the mice were subject to middle cerebral artery occlusion (MCAO) for 1.5 h with the use of an intraluminal filament technique, and then reperfused for 3 h, 12 h, and 24 h, respectively, so as to induce focal cerebral ischemia-reperfusion models. In the sham group, the external carotid artery (ECA) was surgically prepared for the insertion of the filament, but the filament was not inserted. A thermostatically controlled infrared lamp was used during the surgery to maintain body temperature at 37°C. Blood pressure, blood gas, brain temperature, EEG and glucose were monitored.

### Histological analysis

Ten mice from each group were anesthetized with 10% chloral hydrate (i.p. 400 mg/kg) and perfused with 4% cold formaldehydum polymerisatum for 30 min. Then, the mice were executed to get the brains. The brains were further fixed in 4% paraformaldehyde for 24 h, and embedded in paraffin wax. The paraffin-embedded blocks were cut into a series of 5 μm-thick slices, and stained with 0.2% thionine. The hippocampus CA1 region was selected for observation.

### Measurement of cerebral infarct size by TTC staining

After reperfusion, another 14 mice from each group were applied to calculate the infarction volume. The brain was coronally sectioned to 2 mm-thick slices and stained with 1% 2, 3, 5-triphenyl tetrazolium chloride (TTC) in phosphate buffer (0.1 M, pH 7.4) for 30 min at 37°C. These slices were refrigerated in 4% formaldehyde in phosphate buffer for 30 min at 37°C with light-free. A digital camera (Color CCD camera TP-6001A, Topica Inc., Japan) was used to capture the images of these slices. The areas of the infarction region were calculated by a Pathology Image Analysis System (Topica Inc., Japan) and the ratio of the infarction volume to the total slice was noted.

### RNA isolation

The total RNA of the left hippocampus was extracted respectively from 9 mice from each group in TRIzol reagent according to the manufacturer’s instructions. Then, an RNeasy Micro Kit (Qiagen, Valencia, CA) was used to further purify and concentrate RNA. The quality of RNA was evaluated by analyzing the 26S/18S ratio with Bioanalyzer microchip (Agilent, Palo Alto, CA).

### Microarray

Hybridization was performed on a microarray containing 374 cDNAs derived from the cDNA Library (Invitrogen, Cat.1065-025) (Shanghai Biochip co., Ltd, China). Each clone was printed as three duplicate spots on a given chip. A single intensity value for each clone was generated through averaging the quadruplet after smoothing spline normalization. All clones used for production of the microarrays were sequence verified. Six to nine biological replicates for each group were hybridized. The RNA in the sham group was labeled with Cy3 dUTP, and others was labeled with Cy5 dUTP. Then the microarrays were hybridized, washed and dried by airing for scanning. Image files were processed using the Axon GenePix 4000B scanner (Axon, USA) and datasets were prepared according to the routine procedures using ArrayTrack software (FDA, USA).

### Microarray data analysis

After robust multiarray analysis and normalization process by GeneSpring, all experimental data were uploaded to the ArrayTrack system (FDA, USA). A one-way analysis of variance (ANOVA) model and Significance Analysis of Microarrays (SAM) were conducted to compare the means of altered genes among different groups. The value on the mean of altered gene was calculated based on at least three independent microarrays. We set P < 0.05 as the statistical significance cutoff. Genes with a p-value less than 0.05 and a fold change greater than 1.5 were applied for further analysis. The up-regulated or down-regulated differentially expressed gene was defined as the expression level of the gene greater than 1.5-fold or less than 0.5-fold, respectively.

### Pathway analysis

All of the differentially expressed genes were uploaded and mapped to GeneGo database in ArrayTrack system (FDA, USA), which included canonic pathway maps, process networks, gene interactions, and etc. The significance of association between these genes and the canonical pathway was measured in the following 2 ways: 1) a ratio calculated using the number of genes from the dataset that maps the pathway divided by the total number of genes that map to the canonical pathway; 2) a P-value, calculated by Fischer’s Exact Test, determining the probability that the association between the genes and canonical pathway was explained by chance alone. The level of statistical significance was set at P < 0.05. Finally, canonical pathways with a P < 0.05 and a fold change > 1.5 were screened and analyzed.

### Network construction and network analysis

The differentially expressed genes list was uploaded to construct and visualize the representative networks for each group using GeneGo MetaCore™ in ArrayTrack System (FDA, USA). ^.^The networks were generated as the interactions (edges) connecting the input genes (nodes). The edges were derived from the genes which had annotated functional interactions in GeneGo database. Three algorithms were used to analyze the properties of the networks: (1) the direct interaction algorithm to map direct gene-gene interactions; (2) the shortest path algorithm to map the shortest path for interaction between differentially expressed genes; and (3) the analyze algorithm to deduce top scoring processes that are regulated by differentially expressed genes [19]. Only the genes in the upload list were contained in the network.

### Node degree calculation

The node degree means the number of edge linked to the node directly in the network as well as the number of nodes linked to the node. We calculated the degree of node with the equation below:

$$ {C}_i^D=\frac{k_i}{N-1}=\frac{{\displaystyle {\sum}_{i\in \kern0.5em C}{a}_{ij}}}{N-1} $$

Any node *і* in the network, *ki* is the degree of node *i*.

### Similarity among the 3 h, 12 h, and 24 h groups

The Jaccard similarity index was used to measure the degree of association among 3 h, 12 h, and 24 h groups. This index considered the similarity among 3 h, 12 h, and 24 h groups as the number of genes shared divided by the total number of genes present in either one of them. It may be expressed as follows:

$$ \mathrm{J}=\mathrm{C}/\left(\mathrm{A}+\mathrm{B}\hbox{-} \mathrm{C}\right) $$

Where A is the number of genes present at a time point; B is the number of genes present at another time point; and C is the number of genes present at both time points. The number of genes present in either of the diseases is given by A + B − C.

## Results

### Pathway map analysis of altered genes

**Figure 1:**
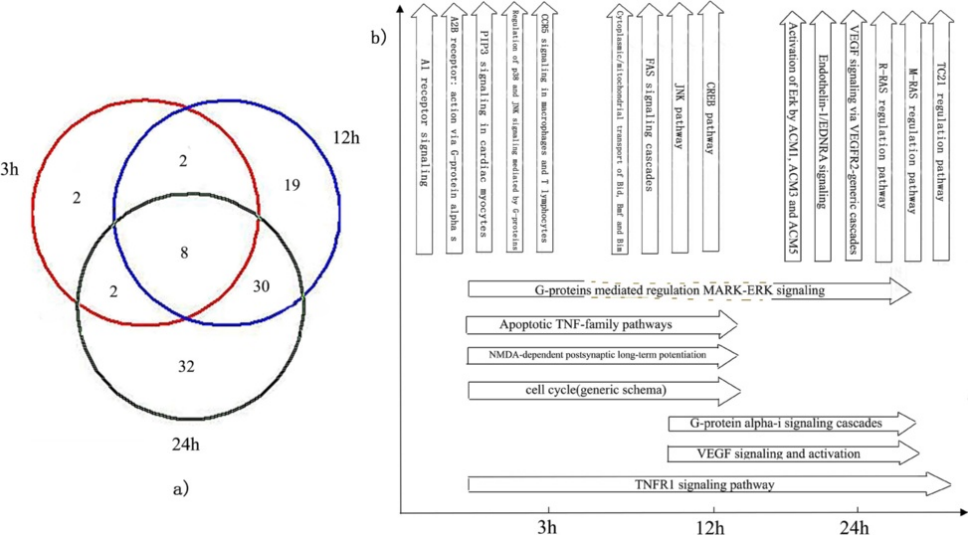
**Top ten pathways in 3 h, 12 h, and 24 h groups after cerebral ischemia-reperfusion injury. (a)** shows the number of overlapping as well as non-overlapping pathways in the 3 h, 12 h, and 24 h groups. The circles in red, blue, and green represent pathways in the 3 h, 12 h, and 24 h groups, respectively. **(b)** shows the names of the top ten pathways at 3 h, 12 h, and 24 h.

After assessing neuronal death at 3 h, 12 h and 24 h after reperfusion (pathological expressions among different groups listed in Additional file 1: Figure S1), the MetaCore™ pathway map analysis of the selected genes was used to identify the statistically significant pathways, based on the calculated P values. The genes activated at 3 h, 12 h, and 24 h are listed in Additional file 2: Table S1. A total of 14, 59, and 72 significantly expressed pathways were identified in the 3 h, 12 h, and 24 h groups, respectively. These included 2, 19, and 32 non-overlapping pathways in the 3 h, 12 h, and 24 h groups, respectively. Eight pathways were overlapped in all the three groups, and several pathways had an overlap between the 3 h and 12h groups (2 pathways), 12 h and 24 h groups (30 pathways), or 3 h and 24 h groups (2 pathways) (Figure 1a).

Amongst the top ten pathways in the 3 h, 12 h, and 24 h groups, 5, 4, and 6 non-overlapping pathways were identified, respectively. Only one overlapping pathway was identified among all the three groups, and several overlapping pathways were detected between the 3 h and 12 h groups (3 pathways), 12 h and 24 h groups (2 pathways), or 3 h and 24 h groups (1 pathway) (Figure 1b).

### The dynamic change of the TNFR1 signaling pathway in 3 h, 12 h, and 24 h groups

**Figure 2:**
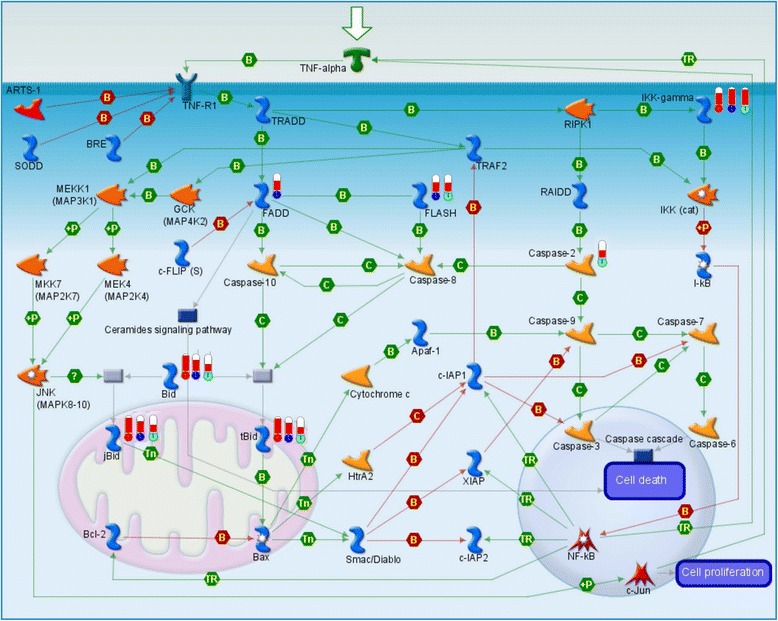
**Change in TNFR1 signaling pathway in 3 h, 12 h, and 24 h groups after cerebral ischemia-reperfusion injury.** Upward thermometers have red color and indicate up-regulated expression levels of the genes. The circles at the base of the thermometers in red, blue, and green represent significantly differential expressions in the 3 h, 12 h, and 24 h groups, respectively.

The TNFR1 signaling pathway was activated across the 3 h, 12 h, and 24 h groups and incidentally, it was the top scoring pathway; however, its constituent genes varied. Across the 3 time points, jBid, tBid, Bid, and IKK-gamma were found to be consistently activated. At 12 h, FADD and FLASH were up-regulated. At 24 h, Caspase-2 and FLASH were up-regulated (Figure 2). Compared with the 3 h group, the 12 h and 24 h groups had more routes to reduce apoptosis response in the TNFR1 signaling pathway. The ceramide signaling pathway, which might reduce cell death, was activated at 12 h, while caspase cascade was activated rapidly at 24 h.

### Process network analysis in 3 h, 12 h, and 24 h groups

**Figure 3:**
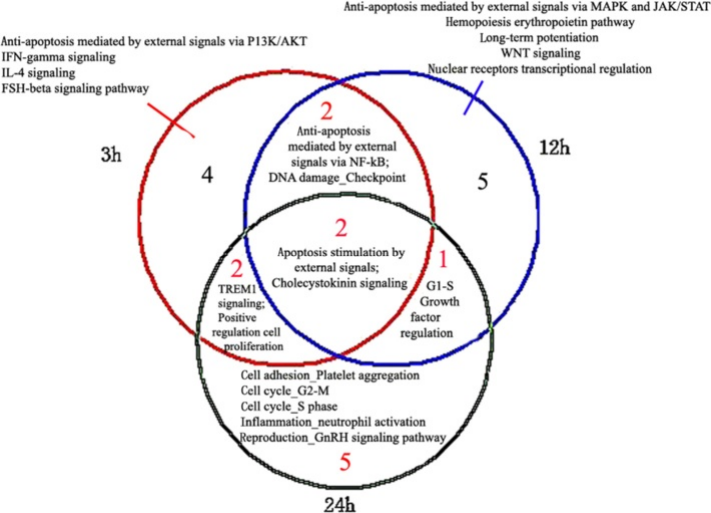
**Top ten process networks in 3 h, 12 h, and 24 h groups after cerebral ischemia-reperfusion injury.** The red, blue, and green circles represent the process networks in the 3 h, 12 h, and 24 h groups, respectively.

The top 10 cellular and molecular processes were defined and annotated by GeneGo. Each process represented a preset network of protein interactions characteristic of the process. A total of 4, 5, and 5 non-overlapping/unique processes were identified in the 3 h, 12 h, and 24 h groups, respectively. Two overlapping processes were identified among the three groups. Two, 1, and 2 processes were found to overlap between the paired groups of 3 h and 12 h, 12 h and 24 h, and 3 h and 24 h, respectively (Figure 3). Among the top 10 cellular and molecular processes, the presence of apoptotic processes was observed in all the 3 groups, while anti-apoptotic processes only existed in the 3 h and 12 h groups, not in the 24 h group.

### The dynamic change in apoptosis stimulation by external signals at 3 h, 12 h, and 24 h

**Figure 4:**
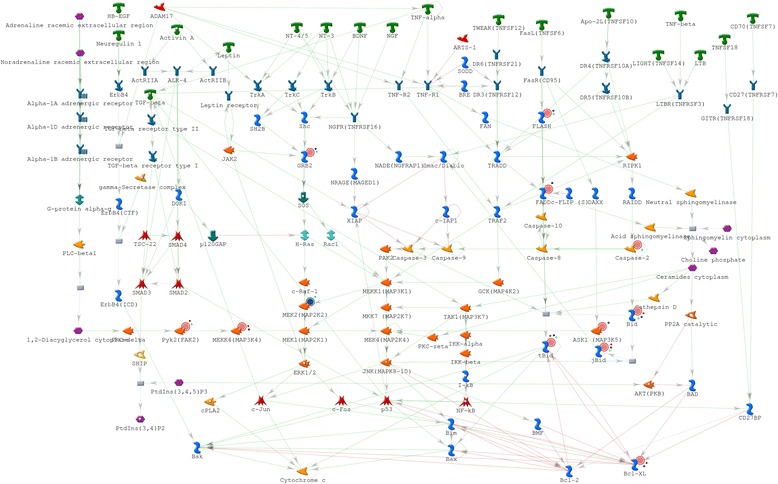
**Changes in process of apoptosis stimulation by external signals in 3 h, 12 h and 24 h groups after cerebral ischemia-reperfusion injury.** The target with red, blue, and green circles represent the process networks in the 3 h, 12 h, and 24 h groups, respectively.

The process of apoptosis stimulation by external signals was consistently activated at all the three time points. It was the top-scored process in the 3 h and 12 h groups, but the fourth-scored process in the 24 h group. Three overlapping differentially expressed genes were identified among the three groups. Two, 2, and 1 overlapping genes were detected in paired groups of 3 h and 12 h, 12 h and 24 h, and 3 h and 24 h, respectively (Figure 4). A total of 0, 2, and 2 non-overlapping differentially expressed genes were identified in the 3 h, 12 h, and 24 h groups, respectively.

### Network analysis in 3 h, 12 h, and 24 h groups

**Figure 5:**
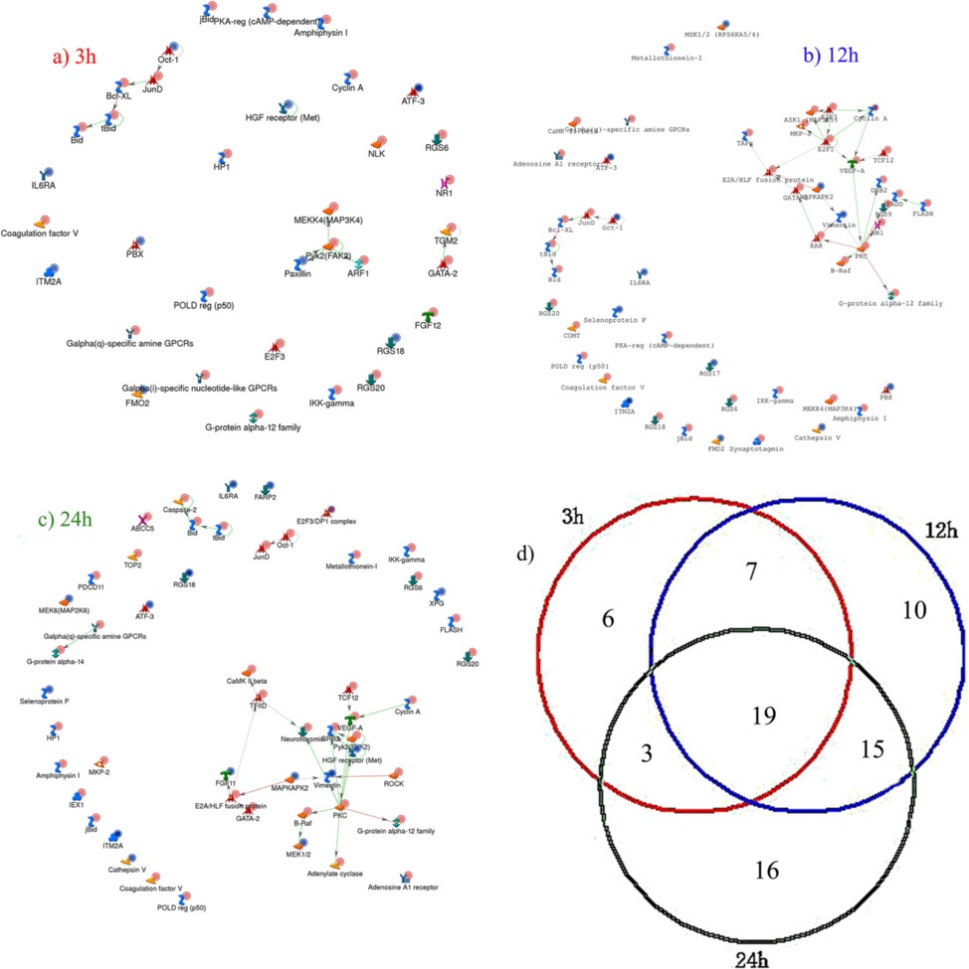
**Networks in 3 h, 12 h, and 24 h groups after cerebral ischemia-reperfusion injury. a)**, **b)**, and **c)** represent the networks in the 3 h, 12 h, and 24 h groups, respectively. **d)** represents the number of overlapping and non-overlapping genes and related molecules in the 3 h, 12 h, and 24 h groups.

In total, 35, 51, 53 genes and related molecules constituted the networks in the 3 h, 12 h, and 24 h groups, respectively. A total of 6, 10, and 16 non-overlapping/unique genes and related molecules were identified in the 3 h, 12 h, and 24 h group networks, respectively. Nineteen overlapping genes and related molecules were identified among the three group networks. Seven, 15, 3 overlapping genes and related molecules were detected between the 3 h and 12 h groups, 12 h and 24 h groups, and 3 h and 24 h groups, respectively (Figure 5).

### Node degree analysis of network

Several nodes were found to be linked with each other. The node whose degree is equal to or greater than 3 is listed in Table 1. Amongst the genes or proteins with a node degree >3, Pyk2, PKC, E2F1, VEGF-A, GATA-2, and Vimentin have already been reported to be related with ischemia-reperfusion injury; however, others such as E2A/HLF fusion protein, TFIID, GRB2, and RAR have not been reported previously.

**Table 1:** **Important gene node list at 3 h, 12 h, and 24 h (degree ≥ 3)**

**Time**	**Node**	**Degree**	**References**
3 h	Pyk2(FAK2)	3	[20]
12 h	PKC	9	[21]
	E2F1	6	[22]
	E2A/HLF fusion protein	4	——
	VEGF-A	4	[23]
	Cyclin A	3	[24]
	E2F3	3	[25]
	Vimentin	3	[26]
	RAR	3	——
24 h	PKC	9	[21]
	E2A/HLF fusion protein	4	——
	VEGF-A	3	[23]
	TFIID	3	——
	GRB2	3	——

### Jaccard similarity analysis of network

The Jaccard similarity index emphasizes the difference of molecular expression at different time points [27]. The Jaccard index for the network level comparison between 3 h and 12 h, 12 h and 24 h, and 3 h and 24 h was calculated to be 0.41, 0.45, and 0.30, respectively, demonstrating a higher level of similarity between 12 h and 24 h than that between 3 h and 12 h. This indicated that the molecular change rate from 3 h to12 h was higher than that from 12 h to 24 h.

## Discussion

To probe the mechanism of ischemia-reperfusion injury, we used the Metacore™ software to analyze the overlapping and non-overlapping pathways as well as cellular and molecular network processes, and reconstructed networks at 3 h, 12 h, and 24 h post injury. The number of activated pathways and cellular and molecular processes at different time points was found to be quite different.

Several common and unique pathways were observed in the 3 h, 12 h, and 24 h groups. A literature review about ischemia-reperfusion injury revealed a close relationship with 21 pathways across time points post injury [28-42], but G-protein signaling_TC21 regulation pathway that was activated at 24 h has not been previously reported to have a direct relationship with ischemia-reperfusion injury. Studies on the TC21 regulation pathway mostly focused on its role in cancer, which mediates its effects via the PI3K-Akt pathway, NF-κB, and cyclin D1 that are all related with cerebral ischemia reperfusion injury [43]. This may be the reason why it has a higher degree in cerebral I-R, and more attention needs to be focused on the role of the TC21 regulation pathway in cerebral I-R.

Changes in six pathways over time were not consistent with those reported in previous studies, including those involved in cytoplasmic/mitochondrial transport of proapoptotic proteins Bid, Bmf, and Bim, FAS signaling cascades, activation of Erk by ACM1, ACM3, and ACM5, VEGF signaling via VEGFR2-generic cascades, MDA-dependent postsynaptic long-term potentiation in CA1 hippocampal neurons, and G-protein mediated regulation of MARK-ERK signaling. In previous studies, these pathways were reported to be expressed at all observed time points [44-48]. The disparity between our findings and previously published data may be resulted from the use of different models, different observed time points, different genes, or different tissues.

**Figure 6:**
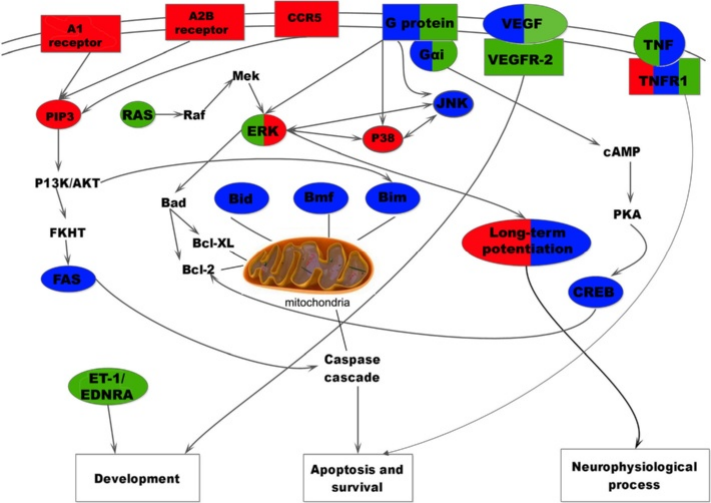
**Visualized dynamic change in the course of pathways at 3 h, 12 h, and 24 h.** Squares filled with color represent the receptor, while ovals represent the pathway. The content not within squares or ovals represents a pathway that is related to a pathway activated in our study. Squares not filled with any color represent a biological function. The red-, blue-, and green-colored squares or ovals represent 3 h, 12 h, and 24 h after cerebral ischemia-reperfusion injury, respectively.

According to enrichment analysis of pathways at different time points, signal transduction, immune response, and apoptosis were identified as the main molecular processes in the 3 h, 12 h, and 24 h groups. However, several differences were observed amongst these three time points. At 3 h, extracellular signals binded to cognate receptors, initiating the immune response. At 12 h, apoptosis was triggered by the differential expression of several pathways, as a result of activation by enzymes and signal transduction. At 24 h, more pathways were activated, especially the G-protein coupled receptor protein signaling pathway (Figure 6).

Process network analysis revealed that apoptotic and anti-apoptotic pathways existed in both 3h and 12h groups, consistent with the findings from a previous study [49]. Our study also found that apoptosis existed in the top 10 processes at 24 h, whereas anti-apoptotic process was absent. Both apoptotic and anti-apoptotic processes might determine the prognosis of injured neurons. Perhaps at 24 h after injury, the anti-apoptotic process was weakened while apoptosis played a leading role, and therefore cell death reached a peak at 24 h post injury. These results demonstrate that early treatment that may activate the process of anti-apoptosis within 12 h has the potential to decrease cell death. This observation is also supported by other study findings. Bcl-xL, belonging to Bcl-2 family, can inhibit cell death [49]. It was differentially expressed at 3 h and 12 h, but not at 24 h, as revealed by analyzing the process of apoptosis stimulation by external signals, which was a pathway common to all three time points. This role of inhibition of anti-apoptotic processes remains to be elucidated.

Based on published literature [50-52], a minority of nodes in a large variety of real world networks is a hub, i.e. a node having a much higher number of neighbors. Hub nodes are important components for shared networks, providing more information than non-hub nodes. The degree is a factor to evaluate the hub node, and a higher degree represents a more important node [53,54]. By analyzing the node degree in the network at different time points, Pyk2 (FAK2) and PKC were identified to be the most important nodes in the 3h, 12h and 24h groups. Pyk2 (FAK2) and PKC, the important proteins in PLC/PKC/Pyk2/Src signaling pathway, can enhance NMDA receptor function in hippocampal neurons [55], and NMDA receptor may lead to Ca^2+^ internal flow, resulting in cerebral ischemic reperfusion [56]. However, other nodes including E2A/HLF fusion protein, TFIID, and GRB2 have never been implicated in the pathogenesis of I-R previously and merit further investigation to understand their functions. Prior studies on E2A/HLF fusion protein mainly focused on its relationship to leukemia; in the context of leukemia, it may induce T-cell apoptosis, and is considered as an very important protein [57]. Besides, TFIID plays a critical role in RNA polymerase II (Pol II) pre-initiation complex (PIC) formation, and therefore, it may affect the process of transcription during cerebral I-R [58]. The relationship between GRB2 and cerebral I-R may be owed to GRB-2-associated binder 1 (Gab1), which is essential in preventing against I-R oxidative injury via mediating survival signaling [59].

Overall, most of earlier studies limited their analysis to a detailed investigation of just a few pathways; while our study provides a comprehensive report of the time course of a differential gene expression profile at 3 h, 12 h, and 24 h post cerebral ischemia injury. Being a method of systematic analysis, it allows for observing changes across 22 different pathways at each time point. Such a method can aid in identifying new important pathways, genes, proteins, or cellular processes by tracking dynamic changes over the course of pathogenesis. One caveat of this method is its limitation in further in-depth study of a specific pathway. Another limitation is that we only observe the changes in gene expression, which may miss post-translational mechanisms. Based on the findings of this study, we propose an in-depth experimental analysis of a few candidate pathways.

## Conclusions

Overlapping and non-overlapping pathways and networks demonstrate the sequential changes in the course of molecular pathways in the hippocampus of ischemic mice. Based on our data, we propose a hypothesis that early treatment which can activate the process of anti-apoptosis within 12 h is essential for decreasing cell death; and this hypothesis should be verified further. To our knowledge, this is the first study to identify one pathway and three genes implicated in cerebral I-R, the roles of which need to be elucidated to understand the pathology of ischemic damages.

## Additional files

Additional file 1: Figure S1: Pathological expressions among different groups. Additional file 2: Table S1: Significantly differential expression genes in the 3 h, 12 h, and 24 h groups.

## Abbreviations

AN: Analyze networks
BBB: Blood–brain barrier
I-R: Ischemia-reperfusion
GK: Glucokinase
GKRP: Glucokinase regulatory protein
ECA: External carotid artery
ANOVA: One-way analysis of variance
SAM: Significance Analysis of Microarrays

## References

[1] Liu B, Zhang Q, Zhang Y, Zhang JX (2007). Study on the changes of pathology and cell apoptosis in different time point following cerebral ischemic reperfusion in rat. Shanxi Med J.

[2] Nakase T, Yoshioka S, Suzuki A (2011). Free radical scavenger, edaravone, reduces the lesion size of lacunar infarction in human brain ischemic stroke. BMC Neurol.

[3] Neumann-Haefelin T, Kastrup A, de Crespigny A, Yenari MA, Ringer T, Sun GH (2000). Serial MRI after transient focal cerebral ischemia in rats: dynamics of tissue injury, blood–brain barrier damage, and edema formation. Stroke.

[4] Reischl S, Li L, Walkinshaw G, Flippin LA, Marti HH, Kunze R (2014). Inhibition of HIF prolyl-4-hydroxylases by FG-4497 reduces brain tissue injury and edema formation during ischemic stroke. PLoS One.

[5] Dohmen C, Kumura E, Rosner G, Heiss WD, Graf R (2005). Extracellular correlates of glutamate toxicity in short-term cerebral ischemia and reperfusion: a direct in vivo comparison between white and gray matter. Brain Res.

[6] Harari OA, Liao JK (2010). NF-κB and innate immunity in ischemic stroke. Ann N Y Acad Sci.

[7] Guo ZH, Li F, Wang WZ (2009). The mechanisms of brain ischemic insult and potential protective interventions. Neurosci Bull.

[8] Vay L, Hernández-Sanmiguel E, Santo-Domingo J, Lobatón CD, Moreno A, Montero M (2007). Modulation of Ca^2+^ release and Ca^2+^ oscillations in HeLa cells and fibroblasts by mitochondrial Ca2 + uniporter stimulation. J Physiol.

[9] Lee JC, Kim IH, Cho GS. Ischemic preconditioning-induced neuroprotection against transient cerebral ischemic damage via attenuating ubiquitin aggregation. J Neurol Sci. 2013. doi: 10.1016/j.jns.2013.10.010. 10.1016/j.jns.2013.10.010 24268923

[10] Bao SY, Shi XQ, Zhang ZL (2014). Changes in expression level of aquaporin-4 during cerebral ischemia-reperfusion injury in rats. Cerebrovasc Dis Foreign Med Sci.

[11] Tang X, Zhong W, Tu Q, Ding B (2014). NADPH oxidase mediates the expression of MMP-9 in cerebral tissue after ischemia reperfusion damage. Neurol Res.

[12] Park JH, Lee CH, Kim IH, Ahn JH, Cho JH, Yan BC (2013). Time-course Changes in Immunoreactivities of Glucokinase and Glucokinase Regulatory Protein in the Gerbil Hippocampus Following Transient Cerebral Ischemia. Neurochem Res.

[13] Stavchanskiĭ VV, Tvorogova TV, Botsina AI, Limborskaia SA, Skvortsova VI, Miasoedov NF (2013). The effect of semax and its C-end peptide PGP on Vegfa gene expression in the rat brain during incomplete global ischemia. Mol Biol (Mosk).

[14] Wang L, Zhou C, Wang Z, Liu J, Jing Z, Zhang Z (2011). Dynamic variation of genes profiles and pathways in the hippocampus of ischemic mice: a genomic study. Brain Res.

[15] GeneGo MetaCoreTM software. Online References: http://www.genego.com and http://www.genego.com/metacore.php.

[16] Baitaluk M, Sedova M, Ray A, Gupta A (2006). Biological Networks: visualization and analysis tool for systems biology. Nucleic Acids Res.

[17] van Leeuwen DM, Pedersen M, Knudsen LE, Bonassi S, Fenech M, Kleinjans JC (2011). Transcriptomic network analysis of micronuclei-related genes: a case study. Mutagenesis.

[18] Hara H, Huang PL, Panahian N, Fishman MC, Moskowitz MA (1996). Reduced brain edema and infarction volume in mice lacking the neuronal isoform of nitric oxide synthase after transient MCA occlusion. J Cereb Blood Flow Metab.

[19] Chen YH, Chen CJ, Yeh S, Lin YN, Wu YC, Hsieh WT (2014). Urethral Dysfunction in Female Mice with Estrogen Receptor β Deficiency. PLoS One.

[20] Liu Y, Zhang GY, Hou XY, Xu TL (2003). Two types of calcium channels regulating activation of proline-rich tyrosine kinase 2 induced by transient brain ischemia in rat hippocampus. Neurosci Lett.

[21] Tang H, Pan CS, Mao XW, Liu YY, Yan L, Zhou CM, et al. Role of NADPH Oxidase in Total Salvianolic Acid Injection Attenuating Ischemia-Reperfusion Impaired Cerebral Microcirculation and Neurons: Implication of AMPK/Akt/PKC. Microcirculation. 2014. doi: 10.1111/micc.12140. [Epub ahead of print]. 10.1111/micc.12140 24702968

[22] MacManus JP, Koch CJ, Jian M, Walker T, Zurakowski B (1999). Decreased brain infarct following focal ischemia in mice lacking the transcription factor E2F1. Neuroreport.

[23] Medvedeva EV, Dmitrieva VG, Povarova OV, Limborska SA, Skvortsova VI, Myasoedov NF (2013). Effect of semax and its C-terminal fragment Pro-Gly-Pro on the expression of VEGF family genes and their receptors in experimental focal ischemia of the rat brain. J Mol Neurosci.

[24] Zhang Q, Chen C, Lü J, Xie M, Pan D, Luo X (2009). Cell cycle inhibition attenuates microglial proliferation and production of IL-1beta, MIP-1alpha, and NO after focal cerebral ischemia in the rat. Glia.

[25] Zhu Y, Jin K, Mao XO, Greenberg DA (2003). Vascular endothelial growth factor promotes proliferation of cortical neuron precursors by regulating E2F expression. FASEB J.

[26] Jiang SX, Slinn J, Aylsworth A, Hou ST (2012). Vimentin participates in microglia activation and neurotoxicity in cerebral ischemia. J Neurochem.

[27] Boyce RL, Ellison PC (2001). Choosing the best similarity index when performing fuzzy set ordination on binary data. J Veg Sci.

[28] Deng C, Luan F, Cruz-Monteagudo M, Borges F, Cordeiro MN (2013). Recent advances on QSAR-based profiling of agonist and antagonist A3 adenosine receptor ligands. Curr Top Med Chem.

[29] Pham N, Dhar A, Khalaj S, Desai K, Taghibiglou C. Down regulation of brain cellular prion protein in an animal model of insulin resistance: Possible implication in increased prevalence of stroke in pre-diabetics/diabetics. Biochem Biophys Res Commun. 2014. [Epub ahead of print]. 10.1016/j.bbrc.2014.04.071 24780399

[30] Hu H, Li Z, Zhu X, Lin R, Peng J, Tao J (2013). GuaLou GuiZhi decoction inhibits LPS-induced microglial cell motility through the MAPK signaling pathway. Int J Mol Med.

[31] Wang J, Li PT, Du H, Hou JC, Li WH, Pan YS (2011). Impact of paracrine signals from brain microvascular endothelial cells on microglial proliferation and migration. Brain Res Bull.

[32] Henshall DC, Engel T (2013). Contribution of apoptosis-associated signaling pathways to epileptogenesis: lessons from Bcl-2 family knockouts. Front Cell Neurosci.

[33] Yin XH, Yan JZ, Hou XY, Wu SL, Zhang GY (2013). Neuroprotection of S-nitrosoglutathione against ischemic injury by down-regulating Fas S-nitrosylation and downstream signaling. Neuroscience.

[34] Zhang Y, Li YW, Wang YX, Zhang HT, Zhang XM, Liang Y (2013). Remifentanil preconditioning alleviating brain damage of cerebral ischemia reperfusion rats by regulating the JNK signal pathway and TNF-α/TNFR1 signal pathway. Mol Biol Rep.

[35] Choi IY, Ju C, Anthony Jalin AM, da Lee I, Prather PL, Kim WK (2013). Activation of cannabinoid CB2 receptor-mediated AMPK/CREB pathway reduces cerebral ischemic injury. Am J Pathol.

[36] Chu LF, Wang WT, Ghanta VK, Lin CH, Chiang YY, Hsueh CM (2008). Ischemic brain cell-derived conditioned medium protects astrocytes against ischemia through GDNF/ERK/NF-kB signaling pathway. Brain Res.

[37] Pyne-Geithman GJ, Nair SG, Stamper DN, Clark JF (2013). Role of bilirubin oxidation products in the pathophysiology of DIND following SAH. Acta Neurochir Suppl.

[38] Kim YR, Kim HN, Ahn SM, Choi YH, Shin HK, Choi BT (2014). Electroacupuncture promotes post-stroke functional recovery via enhancing endogenous neurogenesis in mouse focal cerebral ischemia. PLoS One.

[39] White BC, Sullivan JM, DeGracia DJ, O’Neil BJ, Neumar RW, Grossman LI (2000). Brain ischemia and reperfusion: molecular mechanisms of neuronal injury. J Neurol Sci.

[40] Chen C, Wang W, Xie MJ, Zhu Z, Liu JY, Yu YF (2005). Effect of cell cycle regulation on delayed neuronal death in rat hippocampus after global ischemia. J Histochem Cytochem.

[41] Chen ZB, Zou F, Yuan F (2005). Alterations in Protein expression of NMDA receptor subunits NRZA and NRZB during reperfusion after ischemia in the rat brain. J Apoplexy Nervous Dis.

[42] Deng X, Zhong Y, Gu L, Shen W, Guo J (2013). MiR-21 involve in ERK-mediated upregulation of MMP9 in the rat hippocampus following cerebral ischemia. Brain Res Bull.

[43] Hasan R, Chauhan SS, Sharma R, Ralhan R (2012). siRNA-mediated downregulation of TC21 sensitizes esophageal cancer cells to cisplatin. World J Gastroenterol.

[44] Chen HZ, Chen WM (2007). Expression of genes Bim and JNK signaling transduction pathway in rat brain following cerebral ischemia. China J Mod Med.

[45] Hu JP, Li J, Gao L (2004). Effect of Qi-invigorating and blood-activating therapy on Fas and FasL protein expression in rats with Qi-deficiency and blood stasis syndrome following local cerebral ischemia reperfusion injury. J Anhui TCM Coll.

[46] Wu XW, Yang H, Wu L, Xue RL (2010). Study on relationship between expression of ERK and cell apoptosis. Nei Mongol J Tradition Chinese Med.

[47] Zhang YL, Liu C, Liu XM, Zheng H, Tao Y, Huang QF (2010). Effect on expression of VEGF and VEGFR2 mRNA of Xinnaoshutong Capsule in rat following focal cerebral ischemia reperfusion. Chin J Pathophysiol.

[48] Wang YQ, Li J, Cao H, Li GM, Zeng YM (2006). The expression and role of ERK and JNK in cerebral ischemic preconditioning in gerbil. Chinese Pharmacol Bull.

[49] Wang N, Wu L, Cao Y, Wang Y, Zhang Y (2013). The protective activity of imperatorin in cultured neural cells exposed to hypoxia re-oxygenation injury via anti-apoptosis. Fitoterapia.

[50] Santana-Codina N, Carretero R, Sanz-Pamplona R, Cabrera T, Guney E, Oliva B (2013). A transcriptome-proteome integrated network identifies endoplasmic reticulum thiol oxidoreductase (ERp57) as a hub that mediates bone metastasis. Mol Cell Proteomics.

[51] Luo T, Wu S, Shen X, Li L (2013). Network cluster analysis of protein-protein interaction network identified biomarker for early onset colorectal cancer. Mol Biol Rep.

[52] Zhou Y, Xu J, Liu Y, Li J, Chang C, Xu C (2014). Rat hepatocytes weighted gene co-expression network analysis identifies specific modules and hub genes related to liver regeneration after partial hepatectomy. PLoS One.

[53] Wasserman S, Faust K (1994). Social networks analysis:methods and applications[M].

[54] Freeman LC (1979). Centrality in social networks:L Conceptual clarifieation[J]. Soc Networks.

[55] Yang K, Lei G, Jackson MF, Macdonald JF (2010). The involvement of PACAP/VIP system in the synaptic transmission in the hippocampus. J Mol Neurosci.

[56] Liu X, Hunter C, Chi OZ (2010). Effects of blockade of ionotropic glutamate receptors on blood brain barrier disruption in focal cerebral ischemia. Neurol Sci.

[57] Honda H, Inaba T, Suzuki T, Oda H, Ebihara Y, Tsuiji K (1999). Expression of E2A-HLF chimeric protein induced T-cell apoptosis, B-cell maturation arrest, and development of acute lymphoblastic leukemia. Blood.

[58] Alpern D, Langer D, Ballester B, Le Gras S, Romier C, Mengus G (2014). TAF4, a subunit of transcription factor II D, directs promoter occupancy of nuclear receptor HNF4A during post-natal hepatocyte differentiation. Elife.

[59] Sun L, Chen C, Jiang B, Li Y, Deng Q, Sun M (2014). Grb2-associated binder 1 is essential for cardioprotection against ischemia/reperfusion injury. Basic Res Cardiol.

