# Supplementary material for: Time-dependent variation of pathways and networks in a 24-hour window after cerebral ischemia-reperfusion injury
Source: BMC Syst Biol. 2015 Feb 27;9:11. doi: 10.1186/s12918-015-0152-4 (PMC4355473; doi:10.1186/s12918-015-0152-4)
Supplement: Additional file 1: Figure S1: — Pathological expressions among different groups. [file 12918_2015_152_MOESM1_ESM.docx]

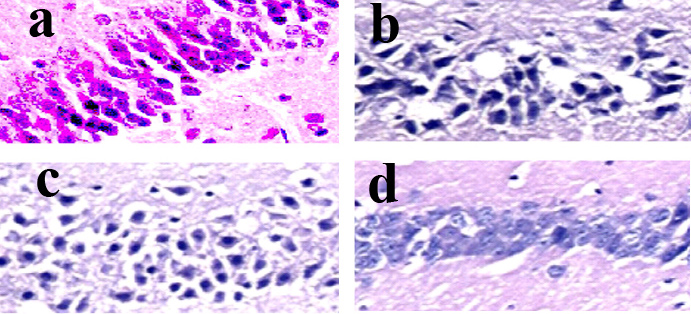


Figure S1 Pathological expressions among different groups.

(a) shows the pyramidal cell of CA1 in the sham group. (b), (c) and (d) show the remaining pyramidal cells of CA1 in the 3h, 12h, and 24h groups. The blue line in picture (e) illustrates the number of neuron counts and the tendency of the remaining neurons in the 3h, 12h, and 24h group.
