# Supplementary material for: Time-dependent variation of pathways and networks in a 24-hour window after cerebral ischemia-reperfusion injury
Source: BMC Syst Biol. 2015 Feb 27;9:11. doi: 10.1186/s12918-015-0152-4 (PMC4355473; doi:10.1186/s12918-015-0152-4)
Supplement: Additional file 2: Table S1: — Significantly differential expression genes in the 3 h, 12 h, and 24 h groups. [file 12918_2015_152_MOESM2_ESM.docx]

Table S1 Significantly differential expression genes in the 3h, 12h, and 24h groups

| Time | Gene | |
| --- | --- | --- |
|  | Up-regulated | **Down-regulated** |
| 3h | Casp8ap2,Ccr3,Cdh11,Eef2k,Lcat,Lta,AV006891,Mmp2,Selenbp2 | ATF3 |
| 12h | Capn9,Fzd10,Tcf3,Tp53i11,Tradd |  |
| 24h | Adamts1,Adcy8,Ankrd6,Cyp51,Daxx,Dgkd,Dusp16,Elk3,Gadd45a,Igfbp2,Il6ra,Nfatc1,Nlk,Pou2f1,Ptk2b,Srf,Tcf12,Tcfe2a,Tgfb2,Wnt3 | Vim,Itm2a |
| 3h,12h | Bak1,Crkl,Gnaq,Pdcd11,Rara,Sox10,Stat6,Za20d1 |  |
| 12h, 24h | Abcc5,Bdnf,Btrc,Cacna1b,Casp2,Dusp4,E2f1,E2f3,Egr1,Fosb,Gab1,Grb2,Htr2c,Il1a,Kcnmb1,Plcg2,Rgs16,Sox30,Stat5a,Vegfa |  |
| 3h,34h | Cbx3,Crem,Hdac1,Nf1,Prkcn,Sos1 |  |
| 3h,12h,24h | Adcy3,Adcyap1r1,Adora1,Ap1m1,Axin,Bad,Barhl1,Bcl2l1,Camk2b,Camk4,Casp2,Casp7,Ccr5,Csf1,D14Abb1e,Dgke,Dkk2,Eif4el3,F5,Fadd,Freq,Gak,Gna12,Gna14,Gpx2,Grin1,Htr1a,Htr1f,Htr3a,Ikbkg,Jund1,Kcnq1,Ldb1,Met,Mlh3,Mogat1,Ngfg,Nkd1,pold2,Ppp2r4,Prkar1b,Pxn,Rgs18,Rgs19,Rgs20,Rgs5,Rgs6,Sh2bpsm1,Shc1,Smad3,Src,Taf7,Tbp,Top2b,Traf2,Wif1 | Map2k5,Mt1 |
